# Heat-Induced Limb Length Asymmetry Has Functional Impact on Weight Bearing in Mouse Hindlimbs

**DOI:** 10.3389/fendo.2018.00289

**Published:** 2018-06-04

**Authors:** Holly L. Racine, Chad A. Meadows, Gabriela Ion, Maria A. Serrat

**Affiliations:** Department of Biomedical Sciences, Joan C. Edwards School of Medicine, Marshall University, Huntington, WV, United States

**Keywords:** limb length discrepancy, endochondral ossification, growth plate, temperature, weight bearing, noninvasive bone lengthening, x-ray, early-onset osteoarthritis

## Abstract

Limb length inequality results from many types of musculoskeletal disorders. Asymmetric weight bearing from a limb length discrepancy of less than 2% can have debilitating consequences such as back problems and early-onset osteoarthritis. Existing treatments include invasive surgeries and/or drug regimens that are often only partially effective. As a noninvasive alternative, we previously developed a once daily limb-heating model using targeted heat on one side of the body for 2 weeks to unilaterally increase bone length by up to 1.5% in growing mice. In this study, we applied heat for 1 week to determine whether these small differences in limb length are functionally significant, assessed by changes in hindlimb weight bearing. We tested the hypothesis that heat-induced limb length asymmetry has a functional impact on weight bearing in mouse hindlimbs. Female 3-week-old C57BL/6 mice (*N* = 12 total) were treated with targeted intermittent heat for 7 days (40 C for 40 min/day). High-resolution x-ray (*N* = 6) and hindlimb weight bearing data (*N* = 8) were acquired at the start and end of the experiments. There were no significant left-right differences in starting tibial length or hindlimb weight bearing. After 1-week heat exposure, tibiae (*t* = 7.7, *p* < 0.001) and femora (*t* = 11.5, *p* < 0.001) were ~1 and 1.4% longer, respectively, on the heat-treated sides (40 C) compared to the non-treated contralateral sides (30 C). Tibial elongation rate was over 6% greater (*t* = 5.19, *p* < 0.001). Hindlimb weight bearing was nearly 20% greater (*t* = 11.9, *p* < 0.001) and significantly correlated with the increase in tibial elongation rate on the heat-treated side (*R*^2^ = 0.82, *p* < 0.01). These results support the hypothesis that even a small limb length discrepancy can cause imbalanced weight distribution in healthy mice. The increase in bone elongation rate generated by localized heat could be a way to equalize limb length and weight bearing asymmetry caused by disease or trauma, leading to new approaches with better outcomes by using heat to lengthen limbs and reduce costly side effects of more invasive interventions.

## Introduction

Unequal length of the lower extremities, or anisomelia (1), has been recognized as a potentially debilitating musculoskeletal condition for over 150 years (2). There are many early reports of left-right asymmetry in limb length caused by fracture/trauma (3–6), tumor (7), infection, or other diseases, particularly during childhood (3, 7–13). The functional outcome is usually pelvic tilt toward the shorter side and compensatory scoliosis of the spine (7, 14, 15), which can lead to gait disturbances and incapacitating back, hip, knee, and/or foot problems (16–25). Kerr et al. (14) were among the first to reveal an association of limb length inequality with back pain when they found that 91% of 150 patients undergoing treatment for low back pain had ≥0.1 cm difference in lengths of their right and left limbs. Rush and Steiner (15) later found that 77% of 1,000 soldiers with low back complaint also had unequal lengths (≥0.1 cm) of their lower extremities. In both studies, the reported left-right differences ranged from 0.1 to >2 cm, making it difficult to ascertain the amount of discrepancy that could cause clinical symptoms.

Most individuals have some degree of normal left-right asymmetry in lengths of their lower limbs (11, 26), although it is often difficult to detect without precise measurement techniques (25, 27–32). Limb length inequality of 2 cm was traditionally used as a clinical threshold for orthopedic intervention, as smaller deviations were not typically considered functionally significant (26, 33, 34). However, there is increasing evidence that weight bearing asymmetry from limb length discrepancies of as little as 0.5–1 cm may cause and/or accelerate the development of knee osteoarthritis and chronic back pain by adulthood (17, 20, 22, 34–41). Moreover, there is evidence that asymmetrical bone density in the femur is associated with asymmetrical gait (42) and osteoporotic fractures resulting from falls can be consequential to limb asymmetry (43), creating an emergent need to identify and correct even small discrepancies in children while their bones are still growing.

For relative comparison, 2 cm is roughly equivalent to a 2.4% difference using a reference limb length of 85 cm for a young adult (total height minus sitting height) (44, 45), while 0.5–1 cm represent less than 0.6 and 1.2% discrepancies, respectively. However, since the same absolute differences may be a larger proportion of total limb length in a child, percentages are a more accurate way to represent the actual discrepancy across the height spectrum (19, 46).

Treatments to correct limb length inequality vary and usually depend on the severity of the discrepancy (47). Shoe lifts are a simple and noninvasive way to functionally treat gait asymmetry, but they do not actually change bone length and are of limited long-term benefit (7, 36, 48, 49). Surgical limb length correction is a century-old method for permanently equalizing limb length. These surgical approaches, which have remained relatively unchanged for decades, involve shortening the longer limb or lengthening the shorter limb through procedures such as epiphysiodesis (temporary or permanent fusion of the growth plate) using staples or screws to halt elongation and/or distraction osteogenesis (Ilizarov technique) using external fixators to lengthen bones (1, 7, 11, 34, 46, 47). The disadvantage of surgical options is that they are invasive, expensive, and carry risks of infection and other potentially major complications (50–52). Less invasive methodologies to correct limb length discrepancies are needed, particularly in light of compelling evidence that even mild limb length inequality (≥1 cm or 1.2% of 85 cm reference limb length) may be linked to the incidence and progression of knee osteoarthritis (22).

Data from our lab and others have shown that chronic exposure to warm ambient temperature increases extremity length in young growing animals [reviewed in Serrat (53)]. Sumner (54) led pioneering research that documented a surprising impact of ambient housing temperature on extremity growth after raising weanling mice at cold and warm temperatures throughout their active growth period. He demonstrated that warm-reared mice had consistently longer ears, limbs, and tails when compared to littermates raised at cooler temperatures (54). This research has since been replicated more recently in a range of vertebrates (53, 55, 56), consistently demonstrating a lengthening effect of heat on limb elements when treatment occurs in an immature animal.

Building on the well-established concept of heat-enhanced limb elongation, we previously developed a noninvasive, once daily unilateral heating model using targeted heat to increase limb length on one side of the body of growing mice without surgical intervention. We used intermittent localized (one-sided) heat because continuous whole body heating does not translate well to a clinical setting. The advantage of unilateral heat is that the non-heated contralateral side can serve as a control. After heat-treating mice for 2 weeks during the active post-weaning growth period, we demonstrated that tibial elongation rate increased >12% on the heat-treated side with accompanying unilateral increases in femoral (1.3%) and tibial (1.5%) lengths (57). Since the most rapid limb elongation occurred during the first week of the experiments, we used the same age mice and heating regime for 1 week in this study to determine whether these relatively small differences in limb length are functionally significant, assessed by changes in hindlimb weight bearing. We used longitudinal x-ray imaging and weight distribution analyses to test the hypothesis that heat-induced limb length asymmetry has a functional impact on weight bearing in mouse hindlimbs. Our ultimate goal is to develop a low-cost, noninvasive method for lengthening bones using targeted heat to help equalize minor deviations in limb length and weight bearing without painful and expensive surgeries.

## Materials and Methods

### Animals and Limb Heating

Procedures for this study were approved by, and carried out in accordance with, guidelines established by the Institutional Animal Care and Use Committee of Marshall University (Protocol 558). A total of *N* = 12 female C57BL/6 mice were obtained from a commercial vendor (Hilltop Lab Animals) at 3 weeks weaning age and housed individually at 21 C as previously described (57). Sample sizes and justification for each measurement below are detailed in the final section of Materials and Methods.

Experiments were carried out between the ages of 3 and 4 weeks from weaning to the pre-pubertal stage (58). This interval was chosen because we have previously found that it is a time of rapid, temperature-sensitive growth in mice (59). By comparison, this period can be considered roughly similar to human development between toddler age and elementary school (pre-teen). Mice were given a single intraperitoneal injection of oxytetracycline (OTC) (7.5 mg/kg, Norbrook 200 mg/ml) at the start of the study to measure tibial elongation rate. OTC is a calcium chelator that becomes incorporated into mineralizing tissue and leaves a band of fluorescence that has long been used to quantify bone elongation rate in growing animals (60–62).

**Figure 1:**
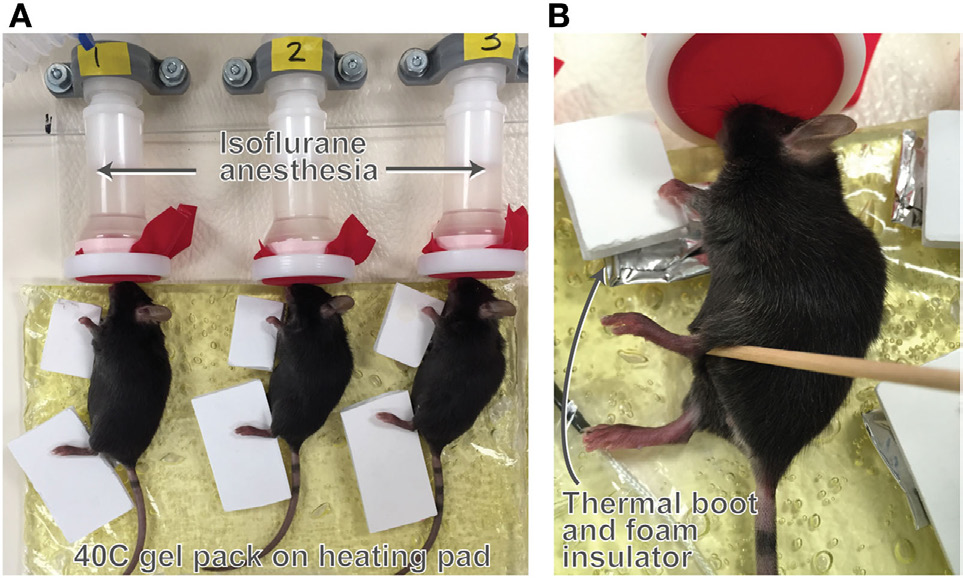
Illustration of the unilateral heating model. Mice were treated with 40 C unilateral heat for 40 min per day for seven consecutive days. Anesthetized mice were placed on gel packs maintained at 40 C atop a heating pad **(A)** so that the entire right side of the body was exposed to heat. The non-treated contralateral side served as the control. Limbs were wrapped in thermal booties to retain heat **(B)** and covered with foam insulators to prevent heat transfer to the non-treated limbs. Core temperature averaged 36 C during the experiments with no major fluctuations. Hindlimb temperature on the heat-treated side averaged 40 C and temperature on the non-treated side was 30 C during treatments as measured by a non-contact infrared thermometer. Procedure room temperature was 18–19 C.

Mice were treated once daily to a 40 C unilateral heating regimen for 40 min each day for 7 days following our published methods (57). Heat treatments were performed at the same time each day near the light cycle start because this is when growth plate height and growth rate are maximal (63, 64). To detail, mice were anesthetized with 1.5% isoflurane and placed in right lateral recumbency on gel packs maintained at 40 C atop a heating pad so that the entire right side of the body was exposed to heat (Figure 1). The non-treated contralateral side served as the control.

Experiments were done in groups of *N* = 6 mice using a 6-position anesthesia manifold (Braintree Scientific) and latex-free band (Thera-band) with small triangular snout openings over the nose cones to prevent anesthesia leakage. Limbs were wrapped in customized foil thermal booties to retain heat and covered with foam insulators to prevent heat transfer to the non-treated limbs (Figure 1). Temperature of the heating pad was continuously monitored using a thermocouple probe. Mouse respiration, core, and skin surface temperatures were recorded every 10 min. We have previously found that core temperature and respiration remain stable throughout the 40-min treatment at a procedure room temperature of 18–19 C (57). Although the entire side of the mouse was heat-treated, we focused on changes in hindlimb length because our previous results have shown that this method of heat application does not impact forelimb (humeral) length but does increase length of the femur, tibia, and hindfoot (57).

### Tibial Radiographs

**Figure 2:**
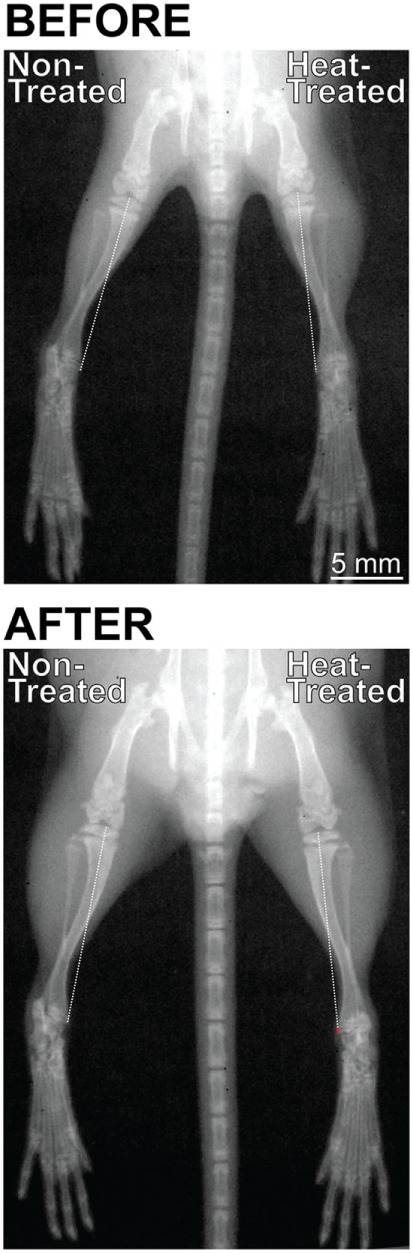
Digital radiographs of the same mouse taken with an IVIS Lumina XRMS live animal imaging system before (3 weeks age) and after (4 weeks age) its right side was subject to daily intermittent limb heating. X-rays were taken in standardized position with the hindlimbs extended and taped down to ensure a true projection of the tibia. Images were rotated so that the spines were aligned at a 90° angle. There were no left-right differences in limb length before the experiments started (top). After 1 week (bottom), the heat-treated right limb was markedly longer than the non-treated left limb as shown by the position of the toes at the bottom of the image. Note that the difference in length is the result of an additive effect of all hindlimb elements. Tibial length was measured from the middle of the proximal articular surface to the distal-most projection of the medial malleolus, shown by the dashed line. The red segment of the line in the right heat-treated limb (bottom) indicates the increase in tibial length relative to the non-treated side (0.16 mm or 1.1% increase in this individual mouse).

X-ray images from *N* = 6 mice were acquired at the start (3 weeks age) and end (4 weeks age) of the limb-heating experiments using an IVIS Lumina XRMS live animal imaging system (Perkin Elmer, Waltham, MA, USA). Mice were individually anesthetized with isoflurane (3.5% induction, graded to 1.5% maintenance) and placed in ventral recumbency on a heated platform. The mouse was secured to the platform with adhesive tape and its hindlimbs were extended and taped down following published methods to ensure reproducible limb positioning and a true projection of the tibia (Figure 2) (65). High-resolution x-ray images were taken following methods described in the literature (66) using the animal energy setting (35 Kv 100 μA, filtered x-rays) at 5 cm × 5 cm field of view with f/stop set at 4 and subject height at 1.5 cm. The mouse was then returned to its individual cage and observed during anesthesia recovery, which typically occurred within 1–2 min.

### Hindlimb Weight Bearing

Starting and ending weight-bearing data were collected from *N* = 8 mice approximately 2 h after the mice had fully recovered from the x-ray procedures. Weight bearing was measured using an incapacitance meter (Stoelting Company, Wood Dale, IL, USA), a testing unit that independently measures weight distribution on the two hindlimbs of a small animal. The incapacitance meter consists of an angled plexiglass chamber that positions mice in a rearing posture so that each hindlimb is placed over a separate force plate that records weight bearing on each side. Mice were first acclimated to the chamber for 2–5 min following published methods (67). Weight bearing was then measured during 3-s intervals and the average weight distributed between the two hindlimbs during that period was recorded, thus accounting for possible shifts in weight from one side to another during testing. A total of 3–5 recordings were taken per mouse per time point and the average weight distributed on each hindlimb was used in data analyses. This is an established method for measuring weight distribution between the hindlimbs and is often used to assess discomfort in models of osteoarthritis and incisional pain (67–71).

### Tissue Collection and Elongation Rate Analyses

Mice were euthanized for tissue harvest 1-day after the last heat-treatment when final x-ray and weight bearing analyses were performed at 4 weeks age. Bone elongation rate was measured in unfixed slab sections of bisected tibiae (*N* = 10). Femora (*N* = 12) were reserved for length measurements. The proximal tibial growth plate was selected to measure elongation rate because it contributes most to tibial lengthening compared to the distal end (72–75) and its relatively flat contour yields a uniform growth rate across the epiphysis and reproducible measurements (61). The distal femoral growth plate was not used because its undulating shape and irregular geometry has a varied growth rate that changes with age (76). The femur was instead saved intact because we have previously found that it can be dissected and cleaned most consistently for reliable bone length data.

To measure elongation rate, one-half of each bisected tibia was placed in a specialized holder on a glass slide and cover-slipped with glycerol in PBS. The other half was reserved for a separate histological study. Fluorescence from the OTC label was visualized at 2.5× magnification using a UV filter on a Leica DM2500 epifluorescence microscope (Leica Microsystems, Wetzlar, Germany) coupled with a QImaging Retiga R6 6.0 megapixel monochrome camera (Surrey, BC, Canada) interfaced to a PC running Ocular Software (version 1.05.211). Images were calibrated in ImageJ software (version 1.48, National Institutes of Health, USA) using a 1-mm stage micrometer. The vertical distance between the metaphyseal chondro-osseous junction and leading (proximal) edge of the OTC band in metaphyseal bone was measured at five equidistant points across the growth plate as detailed in our previous study (57). Measurements were averaged and divided by the 8-day labeling period to estimate daily elongation rate (μm/day).

### Bone Length Measurements

Tibial length at the start and end of the study was measured from digital x-rays calibrated in ImageJ using a razor blade. The tibia was selected for longitudinal bone length data because its landmarks could be most reproducibly identified from a standardized limb position, which is essential when comparing right and left sides of the same animal over time. The femur was not used for longitudinal x-ray data because its proximal and distal landmarks were more prone to distortion. Tibial length was measured from the middle of the proximal articular surface to the distal-most projection of the medial malleolus (see Figure 2).

Femora collected at the study endpoint were dissected, cleaned, dried overnight, and scanned on a flatbed scanner. Scans were calibrated in ImageJ using a metric ruler. Measurements were made from the scanned and calibrated images by drawing a line parallel to the shaft between the proximal-most point on the greater trochanter to the distal-most point on the medial condyle. This scan-based method of bone measurement is an established technique for obtaining limb length data from mice (77, 78).

### Sample Sizes and Statistical Analyses

Two individuals collected measurements separately and observer averages were used. Statistical analyses were performed in SPSS 21.0 software with α = 0.05 as accepted significance. Comparisons between heat-treated and non-treated sides were done using one-tailed paired *t*-tests. One-tailed tests were performed because of the *a priori* hypothesis that the heat-treated side would be larger after treatment. Two-tailed comparisons were used at the start of the study since there was no *a priori* expectation of right-left asymmetry prior to heat-treatment. The relationship between hindlimb weight bearing and tibial elongation rate was assessed using linear regression with asymmetry in tibial elongation rate as the regressor and weight bearing as the dependent variable.

Sample sizes (minimum *N* = 6 mice per variable unless otherwise stated) were determined *a priori* by estimating the effect size and data variability to yield a statistical power of 80% at α = 0.05. The study included a total of *N* = 12 mice to account for potential sample loss. X-ray data were not obtained from half of the mice (*N* = 6) because of technical problems with limb positioning. Other missing data are the result of sample loss due to dissection damage, poor acclimation to the incapacitance meter, and/or improper limb position in the x-ray imager. Missing cases were excluded from statistical testing on an analysis-by-analysis basis. Sample sizes for each analysis are detailed in figure legends and Table S1 in Supplementary Material.

## Results

### Limb-Heating Experiments

Physiological parameters during limb heating were consistent with our prior study (57). Core temperature and respiration were 36 C and 110 breaths/min, respectively, under anesthesia. Hindlimb temperature on the heat-treated side averaged 40 C during treatments. Temperature on the non-treated side was 30 C. The average time to anesthesia recovery after limb heating was 1 min. Mice gained an average of 5 g between the start and end of the study, which parallels the weight gain of age-matched non-treated control mice (57).

### Bone Lengths and Tibial Elongation Rate

**Figure 3:**
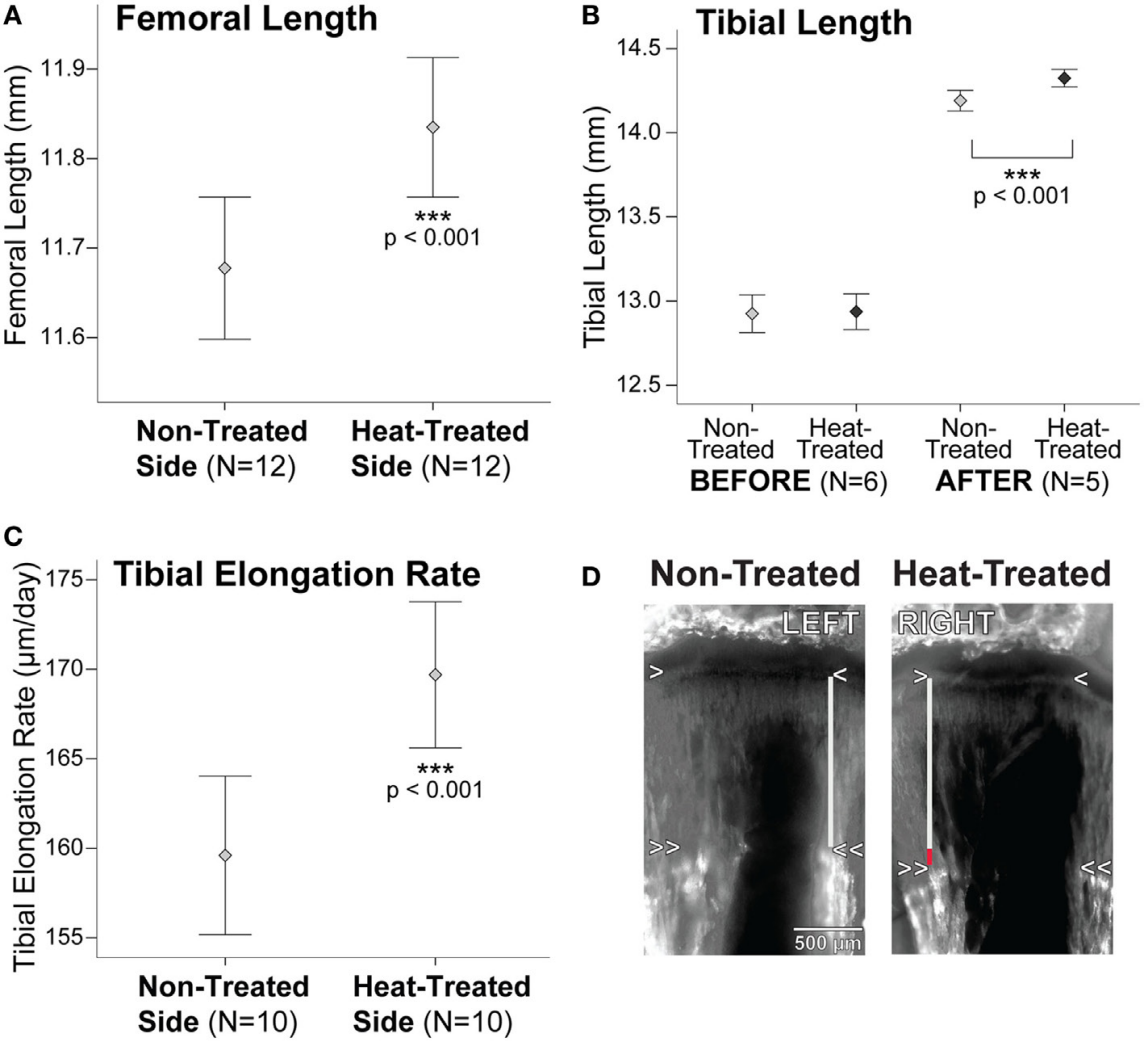
Bone lengths and tibial elongation rate are increased on the heat-treated side. **(A)** Error bar plot shows a 1.4% (0.16 mm) increase in endpoint femoral length on the heat-treated side when compared to the non-treated contralateral side. When tracked longitudinally on x-rays, tibial length **(B)** did not show significant left-right differences before the experiments. After 1-week heat-treatment, tibial length increased nearly 1% (0.13 mm) on the heat-treated side. Femoral length was not measured on x-rays due to inconsistencies in proximal and distal landmarks and was, therefore, only measured from bones dissected and scanned at endpoint (see text). Tibial elongation rate **(C)** increased >6% (10 µm/day) on the heat-treated side. **(D)** Left-right tibial slab sections from the same mouse labeled with oxytetracycline (OTC) show the increase in elongation rate on the heat-treated right side. The metaphyseal chondro-osseous junction is indicated by the single arrowheads. Double arrowheads show the OTC band in metaphyseal bone. Tibial elongation rate was calculated by measuring the vertical distance between the arrowheads (gray lines). The red segment of the vertical line on the heat-treated side shows the total increase in length measured over 8 days. Mean ± 1 SE plotted.

Summary statistics are listed in Table S1 in Supplementary Material. There were no significant right-left differences in starting tibial length measured on x-rays (*t* = 0.65, *p* = 0.55) (Figures 2 and 3B). After 1 week intermittent heat exposure, tibial length measured on x-rays (*t* = 7.7, *p* < 0.001) and endpoint femoral length measured on digitized bone scans (*t* = 11.5, *p* < 0.001) averaged ~1 and 1.4% greater, respectively, on the heat-treated side compared to the non-treated contralateral side (Figures 2 and 3A,B). When animals were tracked individually over time, all mice showed increases in tibial length on the heat-treated side (Figure S1A in Supplementary Material), which averaged 0.13 mm longer than the non-treated side at the endpoint (Table S1 in Supplementary Material). The rate of tibial elongation measured by OTC labeling was over 6% greater on the heat-treated side (Figures 3C,D). The growth acceleration averaged 10 µm/day (paired *t* = 5.19, *p* < 0.001) and the total difference in elongation of the proximal tibia averaged 0.08 mm over the 8-day labeling period.

### Hindlimb Weight Bearing

**Figure 4:**
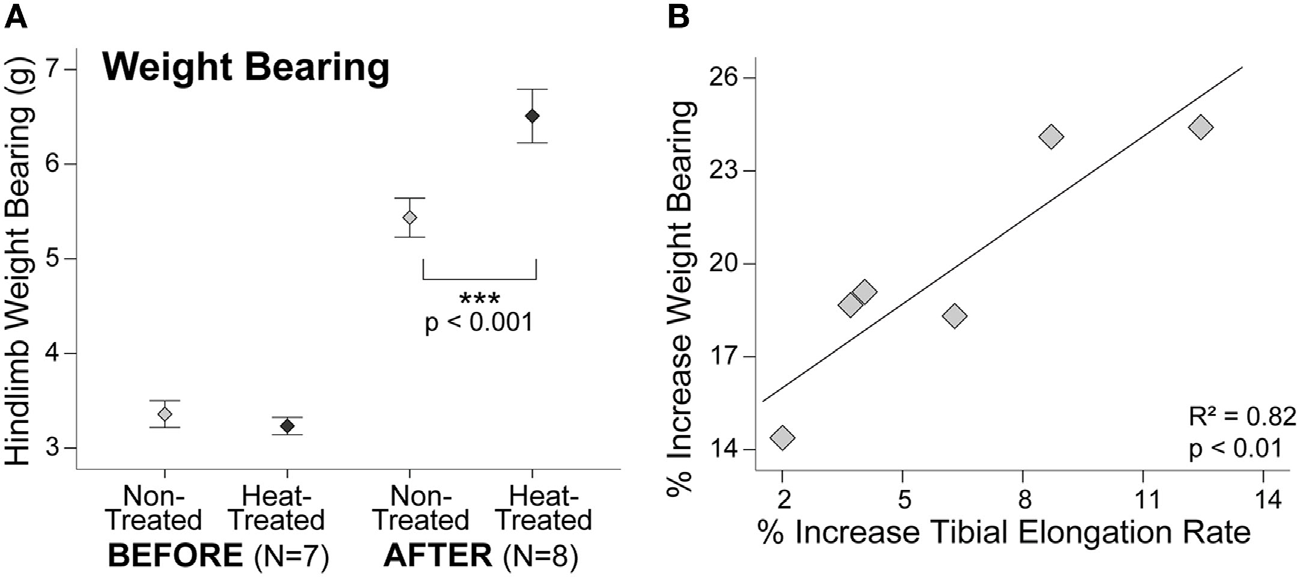
Increases in weight bearing and tibial elongation rate are correlated. Error bar plot shows that hindlimb weight bearing measured with an incapacitance tester **(A)** did not show significant left-right differences before the experiments. After 1-week heat-treatment, hindlimb weight bearing increased nearly 20% (>1 g) on the heat-treated side. Mean ± 1 SE plotted. Scatter plot **(B)** shows a significant positive linear relationship between the increase in weight bearing and increase in tibial elongation rate after 1 week of daily intermittent limb heating (*y* = 14.23 + 0.9*x*). Individual mice are plotted.

Similar to starting tibial length, there were no significant right-left differences in hindlimb weight bearing at the start of the study (*t* = 0.94, *p* = 0.38) (Figure 4A). Average weight bearing was slightly less on the side designated for heat-treatment before the experiments started (Table S1 in Supplementary Material), but this difference was not significant due to the overlapping ranges. By the end of the study, hindlimb weight bearing was nearly 20% greater on the heat-treated side (*t* = 11.9, *p* < 0.001) (Figure 4A). When plotted individually, all mice displayed sharp increases in weight bearing on the heat-treated side (Figure S1B in Supplementary Material). There was a significant positive linear relationship between the increase in weight bearing and increase in tibial elongation rate on the heat-treated side (*R*^2^ = 0.82, *p* < 0.01) (Figure 4B).

## Discussion

The goal of this study was to determine whether relatively small temperature-induced differences in bone length are functionally significant, assessed by changes in hindlimb weight bearing. Our data support the hypothesis that heat-induced limb length asymmetry has a functional impact on weight bearing in mouse hindlimbs without impacting overall body growth.

### Asymmetry in Bone Length and Tibial Elongation Rate

We used longitudinal x-ray imaging to show a 0.13 mm right-left difference, or nearly a 1% increase, in tibial length on the heat-treated side after 1 week of treatment. Although we found a greater percentage increase in femoral length measured at endpoint (1.4%), the difference may be due to the larger sample sizes available for the femur (see Table S1 in Supplementary Material) and/or the difference in methodology since landmarks are more accurate in digitized images of dried mouse bones compared to radiographs. We were not able to measure endpoint tibial length in dried bones because the tibia was sectioned for elongation rate analyses, and we were not able to measure femoral length from x-rays because of problems with distortion of its proximal and distal landmarks (see Materials and Methods). However, the absolute change in tibial length on x-rays is consistent with elongation rate differences observed independently using fluorochrome bone labeling. Growth of the proximal tibia measured in postmortem sections was 0.08 mm greater on the heat-treated side relative to the non-treated side (Figure 3D). This is about 62% of the 0.13 mm difference we found in total tibial length measured using radiographic methods. It is well established that the contributions of proximal and distal growth plates to bone lengthening are not equal. Approximately 57% of overall tibial growth occurs at its proximal end (72–75); however, this varies from 50 to 80% depending on age, as the proximal tibia contributes more to overall lengthening as age increases (75). Our results (62% of growth at proximal end in a 4-week-old mouse) fit well within this expected range.

Although we only quantified percentage increases in femoral and tibial lengths in this study, we have previously shown that all heat-treated hindlimb elements, including the foot, are longer than those on the non-treated side (57). It is important to consider that the additive effect of each limb element will produce a greater impact on total limb length than simply considering percent change of one single bone (see Figure 2). We reported left-right differences in the femur and tibia only because we were not able to accurately measure all limb elements to calculate total hindlimb length using the same methodology. However, we were still able to show that a right-left discrepancy of less than 1.5% in the femur and 1% in the tibia can together cause a nearly 20% imbalance in weight distribution in normal healthy mice. These results suggest that the increase in bone elongation rate generated by targeted heat could be a non-surgical option for equalizing mild limb length and weight bearing inequality in children that could otherwise progress into more significant and chronic musculoskeletal problems such as back pain and early-onset osteoarthritis in adults.

### Hindlimb Weight Bearing

The relatively small limb length asymmetry in our study was associated with a nearly 20% difference in weight bearing in young mice. For comparison, pain studies have shown between 20 and 60% decreases in weight bearing in rats with unilaterally induced osteoarthritis (71, 79). Our results are consistent with experiments that have found changes in gait, muscle exertion, and oxygen consumption with mild (<1–2 cm) limb length inequality in humans (24, 39–41, 80, 81). Our findings (Figure 4B) are also consistent with human studies that have shown gait asymmetry increases as the magnitude of limb length inequality increases (18, 41). These results suggest that targeted heat therapy may offer new possibilities for correcting minor limb length discrepancies and accompanying gait asymmetry in children before orthopedic problems escalate.

One caveat is that we were only able to collect a static measure of weight bearing using an incapacitance meter and we did not perform dynamic gait analyses in our study. Our method involves acclimating mice to an angled plexiglass chamber that places mice in a rearing posture so that each hindlimb is placed over a force plate that records weight bearing on each side. In order to obtain an accurate recording, the mouse must be in a proper position facing forward with both hindlimbs on the force plate and both forelimbs on the angled plexiglass (68). One disadvantage is that the mice are restrained in this type of tester and often become exploratory and/or overly active in the chamber (82). Since data can only be used when the mice are calm, immobile, and properly positioned during testing, we were only able to get reliable longitudinal data (starting and ending weight bearing in same mouse) from a total of *N* = 6 out of the 12 study mice. Another disadvantage is that mice are quadrupeds and static hindlimb weight bearing may not accurately represent the dynamic changes in gait caused by limb length inequality. Collecting future data using a dynamic weight bearing system would be an advantage by allowing us to collect the same data in unrestrained mice in a natural posture without user interference (83, 84). However, the correlation between our weight bearing and limb length asymmetry data (see Figure 4B) still provides strong support for our overall hypothesis.

An additional limitation of our study is that we did not follow these mice to skeletal maturity after the heat-treatments, which is a necessary future study to provide translational relevance. We have previously demonstrated that heat-induced limb length differences do persist at skeletal maturity in 12-week-old mice that were examined 7 weeks after a juvenile heat-treatment (57), suggesting that weight bearing asymmetry would likewise persist. However, it will be important to confirm that the weight bearing effects are still evident in adult animals when the treatment is performed during the juvenile growth period. An important consideration of using a mouse model is that many rodents do not fully fuse their growth plates, allowing a potential for continued skeletal growth in adults. However, rodents do experience a significant age-associated growth decline with concomitant changes in physeal structure and growth factor expression that prevent further lengthening (85–88). Thus although the growth plate may be detected in adult mice, there is little potential for significant limb elongation and heat-enhanced bone growth beyond skeletal maturity. For these reasons, it will be important to identify and treat limb length and weight bearing asymmetry in human children when the growth plate is open and active. We are currently examining growth plate histology in a separate ongoing study in our lab in order to identify mechanisms and potential targets of the heat-enhanced growth effect.

In conclusion, we believe that heat-based methods for treating minor limb length discrepancies may ultimately provide alternatives to traditional surgical approaches that can be painful and invasive. Our results support the hypothesis that even a small bone length discrepancy (less than 1.5%) can cause imbalanced weight distribution (nearly 20%) in healthy mice. The next step is to test this application in larger animal models in an effort to translate the method to humans. Results could eventually lead to new approaches with better outcomes by using our model of targeted heat therapy to reduce costly side effects of surgeries and more invasive interventions. The increase in bone elongation rate generated by localized heat could help equalize minor limb length and weight bearing asymmetry in children caused by a spectrum of linear growth disorders, which could otherwise lead to painful chronic conditions such as scoliosis, back pain, and early-onset osteoarthritis. The major advantage of this noninvasive, straightforward approach is that heat can be used virtually anywhere from urban to rural settings where access to resources might be limited.

## Data Availability

All relevant data generated for this study are included in the manuscript.

## Ethics Statement

Procedures for this study were approved by, and carried out in accordance with, guidelines established by the Institutional Animal Care and Use Committee of Marshall University (Protocol 558).

## Author Contributions

MS and HR designed research, tested equipment, and wrote the paper; MS, HR, CM, and GI performed experiments, edited drafts, and approved the final version of the manuscript; MS and CM collected data; MS analyzed data.

## Conflict of Interest Statement

The authors declare that the research was conducted in the absence of any commercial or financial relationships that could be construed as a potential conflict of interest.
