# Supplementary material for: Heat-Induced Limb Length Asymmetry Has Functional Impact on Weight Bearing in Mouse Hindlimbs
Source: Front Endocrinol (Lausanne). 2018 Jun 4;9:289. doi: 10.3389/fendo.2018.00289 (PMC5994414; doi:10.3389/fendo.2018.00289)
Supplement: Supplementary file 1 [file table_1.docx]

Supplementary Material

**Heat Induced Limb Length Asymmetry Has Functional Impact on Weight Bearing in Mouse Hindlimbs**

**Holly L. Racine, Chad A. Meadows, Gabriela Ion, Maria A. Serrat***

*** Correspondence:** Maria A. Serrat: serrat@marshall.edu

**Supplementary Table 1.** Comparison of non-treated and heat-treated sides.

| Parameter | **Non-Treated**  **(30C)** | **Heat-Treated**  **(40C)** | **Difference** | **Percent Increase** | **N** |
| --- | --- | --- | --- | --- | --- |
|  |  |  |  |  |  |
| **Tibial Length at Start (mm)** | 12.93 (0.27) | 12.94 (0.26) ^ns^ | 0.01 | 0 | 6 |
| **Tibial Length at End (mm)** | 14.19 (0.14) | 14.32 (0.12) ^a^ | 0.13 | 0.9 | 5 |
| **Tibial Elongation Rate (µm/day)** | 159.6 (14.0) | 169.7 (12.9) ^a^ | 10.1 | 6.3 | 10 |
| **Femoral Length (mm)** | 11.68 (0.28) | 11.84 (0.27) ^a^ | 0.16 | 1.4 | 12 |
| **Hindlimb Weight Bearing at Start (g)** | 3.36 (0.38) | 3.23 (0.25) ^ns^ | -0.13 | -4 | 7 |
| **Hindlimb Weight Bearing at End (g)** | 5.44 (0.58) | 6.51 (0.80) ^a^ | 1.07 | 19.7 | 8 |
|  |  |  |  |  |  |

Values are mean (standard deviation). Sample size (N) is number of left-right pairs. Difference and percent increase are calculated relative to heat-treated side. Significantly larger on heat-treated side by one-tailed paired t-test (two-tailed at start): ^a^ p < 0.001; ^ns^ non-significant.


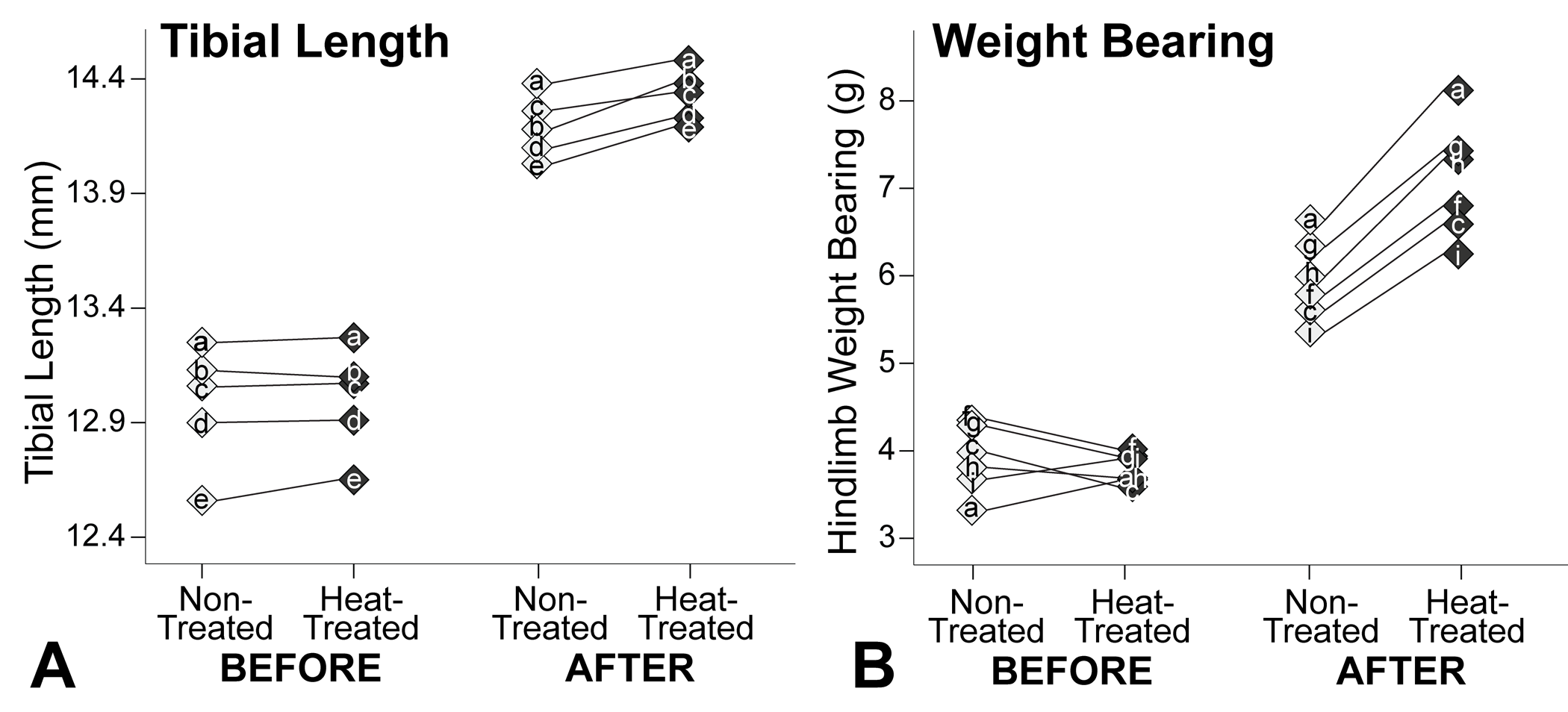


**Supplementary Figure 1.** Increases in tibial length and hindlimb weight bearing in individual mice after one week of unilateral limb heating. Plots of left-right differences in tibial length (A) and hindlimb weight bearing (B) before and after limb heating experiments show individual variation in the measurements. There were no significant left-right differences in either variable at the start of the study. After one-week heat treatment, all mice showed increases in tibial length and hindlimb weight bearing on the heat-treated side. Letters represent individual mice, which were included in the group means shown in Figures 3-4 of the main text.
